# Targeting splicing for hematological malignancies therapy

**DOI:** 10.1186/s12864-024-10975-y

**Published:** 2024-11-11

**Authors:** Monika Szelest, Krzysztof Giannopoulos

**Affiliations:** Department of Experimental Hematooncology, Medical University of Lublin, Chodzki 1, Lublin, 20-093 Poland

**Keywords:** Splicing, Antisense oligonucleotides, Therapy resistance, Leukemia, Splicing modulators

## Abstract

Alterations in splicing patterns of leukemic cells have a functional impact and influence most cellular processes since aberrantly spliced isoforms can provide a proliferative advantage, enable to evade apoptosis, induce metabolic reprogramming, change cell signaling and antitumor immune response, or develop drug resistance. In this Review, we first characterize the general mechanism of mRNA processing regulation with a focus on the role of splicing factors, which are commonly mutated in blood neoplasms. Next, we provide a comprehensive summary on the current understanding of alternative splicing events, which confer resistance to targeted treatment strategies and immunotherapy. We introduce the functional consequences of mis-spliced variants (*CD19*-∆ex2, *CD22*-∆ex2, *CD22*-∆ex5-6, *CD33*-∆ex2, *PIK3CD-S*, *BCR-ABL*^35INS^, *BIM-γ*, *FPGS-8PR*, *dCK*-∆ex2-3, and *SLC29A1*-∆ex13) production in leukemic cells. Of therapeutic relevance, we summarize novel strategies focused on pharmacological correction of aberrant splicing, including small-molecule splicing modulators and splice-switching oligonucleotides. We also include the findings of recent preclinical investigation of the antisense strategies based on modified oligonucleotides. Finally, we discuss the potential of emerging combination therapies for the treatment of hematological disorders with disrupted splicing.

## Background

Alternative splicing (AS) is an essential nuclear process that generates multiple distinct isoforms from a single precursor mRNA transcript via removal of introns and ligation of exons. Nearly all multi-exon human genes undergo AS, and this process has been indicated as a major source of cellular protein diversity. Besides regulation of gene expression, AS is involved in mRNA translation and mRNA quality control through nonsense-mediated decay (NMD). Depending on the type of splicing event, the produced transcripts might differ in structure and function, including stability, localization, and translation efficiency. AS patterns are categorized into alternative 3’ and 5’ splice site selection, exon skipping, mutually exclusive exons, and intron retention [1].

**Fig. 1:**
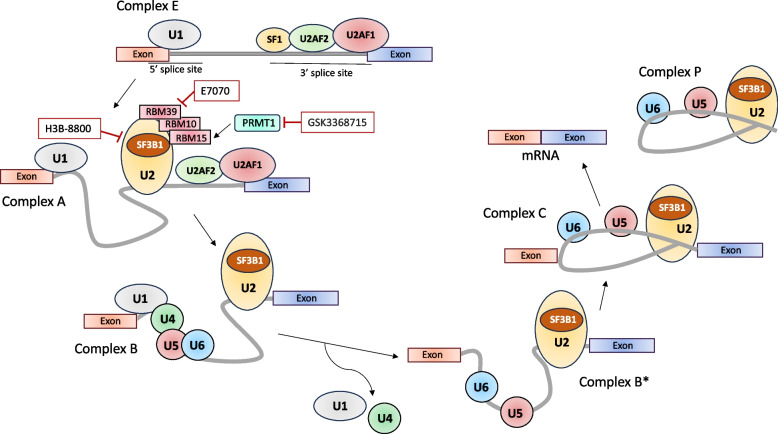
Spliceosome assembly. Five small nuclear ribonucleoproteins (snRNPs) and various auxiliary proteins interact with pre-mRNA in a specific order to form the spliceosome. The U1 snRNP is recruited to the 5’ splice site, whereas splicing factor 1 (SF1), U2 auxiliary factor 2 and 1 (U2AF2 and U2AF1) interact with the branchpoint sequence, the polypyrimidine tract, and the AG dinucleotide of the 3’ splice site, respectively, thus forming complex E. Then, the U2 snRNP displaces SF1 and stably associates with the branch site itself, leading to the formation of complex A. SF3B1 is a U2 snRNP component that facilitates the identification of the intron branch site by U2 snRNA. RNA-binding motif proteins RBM39, RBM10, and RBM15 recognize distinct regulatory sequences located in introns and exons, thus modulating the identification of the neighboring 3’ splice site. Subsequently, the U4/U6.U5 tri-snRNP is recruited, generating complex B. After several rearrangements, followed by the removal of U1 and U4 snRNPs, the catalytically active conformations of the spliceosome are formed, known as complex B*, which carry out the first transesterification reaction. This yields the C complex that catalyzes the second splicing step, which enables lariat removal and exons ligation. H3B-8800, E7070, and GSK3368715 are inhibitors of spliceosome machinery components SF3B1, RBM39, and PRMT1, respectively, which clinical efficiency is being evaluated in clinical trials

The splicing process undergoes sequential reactions, which require the recruitment of multiple ribonucleoprotein complexes (snRNPs) and splicing factors (SFs) (Fig. 1).

Furthermore, the splicing specificity is modulated by different *cis*-regulatory elements, which are classified as exonic or intronic splicing enhancers (ESE/ISE) or silencer sequences (ISS/ESS). These *cis*-elements interact with trans-acting SFs to select the splice site or induce/suppress the spliceosome assembly [1]. Although the precise regulation of AS events is crucial to determine tissue types, various aberrantly spliced isoforms are frequently observed in several tumors, indicating a common regulatory network of this process among distinct tissues [2].

Cancer-related splicing events can be implicated in the development and progression of disease, including cell proliferation, apoptosis, invasion, metastasis, evasion of immune surveillance, and chemo- or radiotherapeutic resistance. In hematological malignancies, AS is commonly disrupted, which is due to recurrent mutations in genes encoding SFs and *trans*-acting elements, *cis*-acting somatic mutations, changed expression of essential splicing regulators, or alterations that indirectly affect AS, such as mutations in chromatin modifiers or transcription factors [3–5].

The most commonly mutated SFs across hematologic malignancies are *SF3B1*, *U2AF1*, and *SRSF2*. Interestingly, mutations in these genes have distinct prognostic impacts in patients with leukemia. A comprehensive RNA-seq analysis of CD34 + cells from patients with myelodysplastic syndromes (MDS) harboring these SFs mutations revealed > 200 disrupted splicing events for each mutated SF and over 100 genes were found to be aberrantly spliced [6]. Interestingly, distinct SF mutations were identified to promote different types of splicing events. For example, *SF3B1* mutations were associated with increased frequency of alternative 3’ splice site selection and intron retention events, while *SRSF2* as well as *U2AF1* mutations were related to a higher proportion of exon skipping [6].

Furthermore, mutations in *SRSF2* and *U2AF1* affecting MDS cells were recently found to promote the formation of R-loops, resulting from the invasion of nascent RNA into template DNA [7, 8]. R-loops are implicated in replication stress and induce the activation of the ataxia teleangiectasia and Rad3-related protein (ATR) pathway.

Nevertheless, not only genetic alterations are responsible for splicing aberrations. Recent studies revealed that intronic polyadenylation (IPA) disruption contributes to a widespread upregulation of truncated mRNA and proteins in malignant B cells collected from patients with chronic lymphocytic leukemia (CLL) [9]. Truncated isoforms generated by aberrant IPA were predominantly produced from tumor-suppressor genes, including *DICER*, *FOXN3*, *CARD11*, *MGA*, and *CHST11 *[9].

Changes in the pre-mRNA processing, such as intron retention, IPA, or generation of fusion transcripts lead to the production of neoantigens generated by cancer-specific mRNAs, which might serve as targets for T-cell based adoptive therapies and cancer vaccines [10]. Furthermore, disruption of large-scale splicing events, such as intron retention and juxtaposition of exons not normally spliced together, might be a source of immunogenic neopeptides derived from abundant mRNAs [11]. Therefore, profiling of effects of abundant splicing might provide novel biomarkers for targeted therapies.

In this Review, we outline in detail the latest studies focusing on the role of AS changes in the context of anti-cancer treatment resistance, as well as opportunities for the development of novel therapeutic approaches.

## AS and response to leukemia treatment

**Fig. 2:**
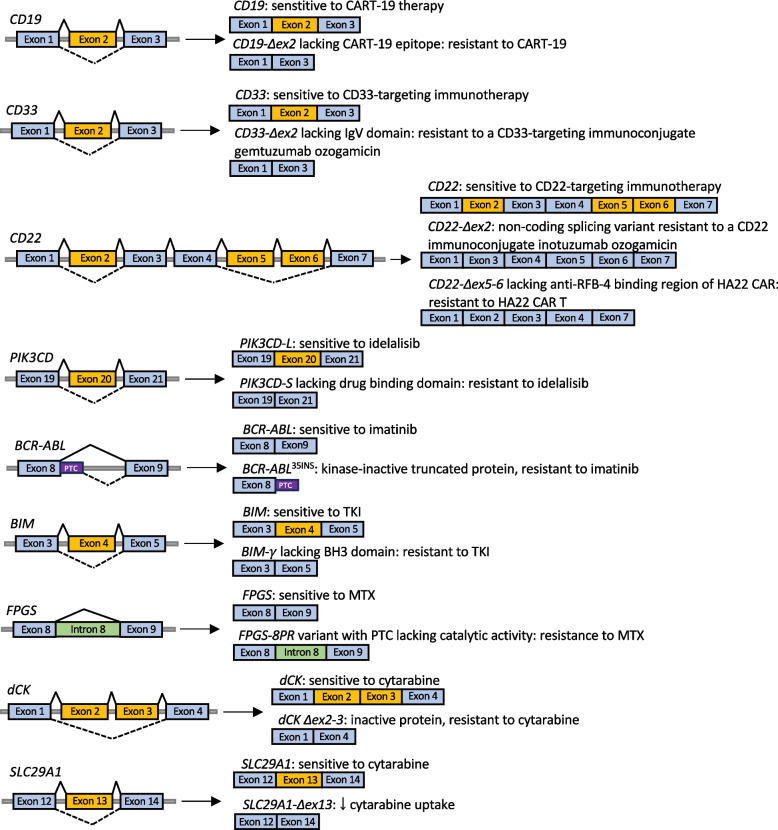
Splice variants conferring resistance to therapy in leukemic malignancies. PTC – premature termination codon, 8PR – partial retention of intron 8, MTX – methotrexate

Emerging evidence from transcriptome studies indicates that mutations in SFs, splicing auxiliary proteins, and epigenetic regulators might drive leukemia development through coordinated effects on the epigenetic state and AS (reviewed elsewhere [5]). Besides leukemogenesis, aberrant splicing events were reported to be implicated in the clinical response to anticancer therapy in hematological malignancies. To date, several AS-related mechanisms that confer resistance to treatment in leukemic cells have been characterized (Fig. 2).

### AS of *CD19*, *CD22*, and *CD33* and immunotherapy evasion

Immunotherapy by chimeric antigen receptor-modified T (CART) cells targeting CD19 has demonstrated an improved clinical efficacy for relapsed and/or refractory B-cell acute lymphoblastic leukemias (B-ALLs) and high-grade lymphomas (HGL). However, over 10% of the pediatric B-ALL patients exhibit antigen loss, which causes resistance to the CD19-directed immunotherapy [12]. Interestingly, it was reported that *SRSF3*-induced AS of *CD19* might contribute to the loss of surface expression of CD19 epitopes in B-ALL patients [13]. Sotillo et al. [13] revealed that downregulation of *SRSF3* in cells induces skipping of *CD19* exon 2, leading to the synthesis of truncated *CD19* isoform (*CD19*-∆ex2), which fails to trigger killing by CART targeting CD19 epitope. Therefore, AS-derived differential CD19 ectodomains might serve as targets for development a novel CART-19, thus improving survival of B-ALL patients.

Furthermore, a recent study Zheng et al. [14] demonstrated a significant role of differential splicing of *CD22* in *de novo* and acquired resistance to CD22-directed immunotherapies in B-ALL. The CD22 expression level was found to be modulated by either inclusion or skipping of the AUG-containing *CD22* exon 2. In vitro experiments revealed that exon 2 skipping results in the production of noncoding *CD22*-∆ex2 splicing variant and conferred resistance to a CD22 immunoconjugate inotuzumab ozogamicin [14]. In addition to *CD22*-∆ex2, *CD22* isoforms lacking exons 5 to 6 (*CD22*-∆ex5-6) were identified in this study. Interestingly, these skipped *CD22* exons 5 to 6 encode the binding region of RFB-4 antibody, which constitutes the basis of HA22 CAR, and, indeed, B-ALL cell lines expressing this isoform of *CD22* were found to be resistant to HA22 CAR T.

Moreover, previous studies identified a specific CD33 polymorphism in the ESE region that modulates the expression of aberrant *CD33* variant lacking exon 2 in acute myeloid leukemia (AML) samples [15]. Notably, *CD33* exon 2 exclusion results in the loss of IgV domain, which is the antibody-binding site for a CD33-targeted immunoconjugate gemtuzumab ozogamicin [15].

### AS of *PIK3CD* and idelalisib resistance

The phosphatidylinositol 3-kinase catalytic subunit p110d (PI3Kd) selective inhibitor idelalisib is used for the treatment of relapsed indolent B-cell malignancies, such as CLL, small lymphocytic lymphoma, and follicular B-cell non-Hodgkin lymphoma [16]. Despite good clinical efficacy, over 20% of patients with CLL have poor response to idelalisib [17, 18]. Although limited data are available on mechanisms of resistance to PI3K inhibitors, it has been demonstrated that AS of *PIK3CD*, resulting in the production of truncated PI3Kd isoform (*PIK3CD-S*), might contribute to a more aggressive oncogenic phenotype. AS-induced exclusion of *PIK3CD-S* exon 20 affects a crucial region of the catalytic domain of the PI3Kd isoform, thus preventing its interaction with idelalisib [19]. Therefore, further experimentation is needed to evaluate whether increased expression of the PI3Kd short isoform is implicated in idelalisib resistance in hematologic neoplasms.

### AS and imatinib resistance in CML

Imatinib is a specific inhibitor of the BCR-ABL1 tyrosine kinase and has proven highly effective in chronic myelogenous leukemia (CML) treatment [20]. Nevertheless, up to 20% of CML patients eventually develop resistance to imatinib [21]. While the most frequent cause of treatment failure with imatinib is a point mutation in *ABL1* kinase domain of *BCR-ABL1*, recent evidence implies that differentially spliced *BCR-ABL1*^35INS^ variant might associated with imatinib resistance in CML [22–25]. An intronic inclusion between *BCR-ABL1*^35INS^ exons 8 and 9 results in a frameshift and truncated version of the encoded protein, which lacks numerous native kinase residues. Furthermore, it has been suggested that these changes induce global conformational movements, thus altering imatinib binding and, in turn, providing resistance to tyrosine kinase inhibitor (TKI) in an expression-dependent manner [23]. Despite the common presence of the *BCR-ABL1*^35INS^ splice variant in imatinib-resistant patients, its biological and clinical significance remains to be clarified.

Moreover, a recent study documented that AS of *BIM* (*BCL2L11*) might affect TKI sensitivity of CML [26]. Among the major *BIM* AS variants, *BIM-γ* is of particular interest, as it lacks the exon-4 encoded BH3 domain, which is required for the apoptotic function of BIM. TKI exposure induces an exon 4-to-exon 3 switch in CML cells containing an intronic deletion polymorphism. The preferential inclusion of exon 3 over exon 4 results in premature termination of translation, thereby leading to the pro-apoptotic BH3 domain loss. These data indicate that the expression of BIM-γ impairs the imatinib-induced apoptosis in CML, thus might contribute to TKI therapy resistance. Nevertheless, it has been shown that this effect could be overcome with BH3-mimetic drugs or splice-switching *BIM* antisense oligonucleotides [26, 27].

Interestingly, drug resistance in hematologic malignancies can be associated with the activity of splicing regulators. For instance, it has been demonstrated that *SRSF1* expression levels in bone marrow samples from chronic phase (CP) CML patients correlate with poorer imatinib responses [28]. Notably, recent RNA-seq data have identified a novel *BCR-ABL1*-independent mechanism, which might contribute to imatinib sensitivity in primary CD34 + progenitors and CML cell lines. It has been demonstrated that the cytokine-mediated SRSF1 upregulation impairs imatinib sensitivity in CP progenitor cells due to the activation of protein kinase C and phospholipase C signaling, which enables enhanced survival and persistence [28]. Thus, highly selective PKC? inhibitors are required to overcome the SRSF1-dependent imatinib resistance in CML patients.

### AS and methotrexate resistance in ALL

To date, methotrexate (MTX) efficacy for childhood ALL has been proven in numerous clinical trials. Furthermore, protocols with high-dose MTX therapy have been proposed for the treatment of adult ALL [29]. However, it has been revealed that aberrant splicing of folypoly-γ-glutamate synthetase (FPGS) contributes to MTX resistance of leukemia cell line models and adult ALL samples [30]. FPGS is a key enzyme responsible for polyglutamylation and subsequent intracellular retention of folates and antifolates, including MTX. Stark et al. [30] indicated that differential splicing of *FPGS* in ALL cells is based on intron retention and/or exon skipping, and results in a loss of FPGS function due to premature termination of translation. Further research identified an intron 8 partial retention as the most prominent *FGPS* splicing alteration in pediatric ALL [31]. This splicing event introduces a premature stop codon of *FPGS*, and thereby results in a loss of a domain responsible for polyglutamylation reaction. Aberrant splicing-related low activity of the enzyme is associated with a very poor response to MTX [29].

### AS and cytarabine resistance in AML

Aberrant mRNA processing was found to be correlated with resistance to cytarabine, an antimetabolite which is frequently used in the treatment of leukemias and lymphomas. Cytarabine activation is catalyzed by deoxycytidine kinase (dCK) and early studies suggested that mutational inactivation of this intracellular enzyme is associated with resistance to cytarabine. However, Veuger et al. [32] observed exon skipping events that generate alternatively spliced *dCK* isoforms in patients with cytarabine-resistant AML. Interestingly, these isoforms were not found in patients with sensitive AML and non-malignant samples.

Cai et al. [33] identified two distinct splicing-related mechanisms of cytarabine resistance in leukemic cells, which were developed through continuous exposure of CCRF-CEM (ALL) cells to the drug. In the first case, high cytarabine resistance of CCRF-CEM clone was due to the production of inactive *dCK* isoforms caused by independent intronic mutations that impair the splicing of exon 2 and exon 3. The second mechanism was observed in a different CRCF-CEM clone, which displayed a moderate resistance to cytarabine. Here, a complex intronic mutation in a gene encoding a member of a nucleoside transporter proteins family, solute carrier family 29 member 1 (*SLC29A1*), disrupted splicing of exon 13 of this gene, and decreased efficiency of intracellular cytarabine uptake.

A recent study by Morales et al. [34] revealed that the development of cytarabine-resistant AML is accompanied by altered phosphorylation of SR proteins, including SRRM2, SCAF1, and U2AF2. These posttranslational modifications of SR proteins might influence their activity in AS processes for various targets, such as the SRRM2 pro-apoptotic targets *BAX*, *CASP3*, and the U2AF1 target *H2AFY* and *WAC* [34]. Of note, the combination of a splicing inhibitor H3B-8800 and BCL2 inhibitor venetoclax significantly improves the therapeutic response in both cytarabine-sensitive and -resistant cells collected from AML patients [34].

### AS and glucocorticoids (GCs) resistance in ALL

Although many improvements in the treatment of pediatric ALL have been achieved over the past years [35], about 20% of patients experience a relapse, mainly due to the development of resistance of leukemic cells [36]. GCs, in particular dexamethasone and prednisone, play an essential role in the chemotherapy regimens for ALL. Despite variable response to GC among individuals with ALL, steroid resistance has been found in a relatively high proportion of patients undergoing induction therapy [37]. Furthermore, blasts collected from relapsed patients displayed increased GC resistance in comparison to leukemic cells isolated at diagnosis [38].

Previous studies indicated that GC resistance is associated with the impaired activity of the GC receptor (GR), resulting from mutations and decreased ligand and/or DNA binding capacity [39]. Furthermore, alternative processing of the *GR* gene results in the production of functionally different receptor subtypes, some of which (e.g. GR-??, GR-??, GR-A, GR-P) acts as a dominant negative inhibitor, thus antagonizing the activity of canonical isoform GR-?? [40]. Interestingly, the increased levels of GR-?? have been correlated with GC resistance in ALL and CLL [41]. However, a recent study by Sciarrillo et al. [42] showed a differential splicing pattern of genes involved in direct response to GCs (*HSP90AA1*, *SGK1*), regulation of proliferation and apoptosis (*TP53*, *BAX*), mRNA splicing (*U2AF1*, distinct *HNRNP*s) as well as energy metabolism (*SOD1*, *SOD2*) in GC resistant primary ALL patient samples.

## Splicing-based therapy approaches

### Splicing modulators targeting SF3B1

Since it has been documented that AS is involved in oncogenesis and disease progression, spliceosome components and auxiliary proteins have become an attractive target for the treatment of several cancer types, including hematological malignancies. In the past decade, numerous pre-clinical studies demonstrated potent antitumor activity of SF3B1-targeting splicing modulators that share a common pharmacophore, including bacterial natural products derived from fermentation of *Pseudomonas* sp. or distinct species of *Streptomyces*, and synthetic compounds. The three families of SF3b-targeting spliceosome modulators comprise spliceostatins (e.g. spliceostatin A, meayamycin, and sudemycins), pladienolides (e.g. FD-895, E7107, H3B-880), and herboxidienes (e.g. GEX and RQN-18690 A) [43]. Notably, tumors exhibiting molecular addiction to weak spliced isoform arising from non-canonical splicing motifs are particularly attractive for the application of these agents [44].

Molecular analyses revealed SF3b-targeting spliceosome modulators interact directly with the HEAT repeats domain of SF3B1 and prevent binding of the U2 snRNA to the pre-mRNA. This results in altered conformation of the protein and impaired recognition of the branchpoint site (BPS), thereby leading to cryptic 3’ splice site selection [45, 46]. Cryogenic electron microscopy structure of the SF3b subcomplexes revealed that the pladienolide B-derived spliceosome modulator E7107 binds in the branch point adenosine-binding pocket and interacts with the key residues of SF3B1 and PHF5A. Notably, the observed binding mode is consistent with previously reported resistance mutations to E7107, such as *SF3B1*^R1074H^ and *PHF5A*^Y36C^[47, 48].

Recently, Crews et al. [49] demonstrated that pharmacological splicing modulation with 17S-FD-895 enables reversion of the pro-survival splicing patterns associated with secondary AML (sAML) and MDS progenitors and impairs leukemia stem cell (LSC) maintenance while sparing normal hematopoietic cells in xenograft mouse models. Notably, comparative whole transcriptome analyses demonstrated that sAML LSC promote expression of pro-survival *BCL2L1* isoform, while aging human progenitors favor production of short pro-apoptotic *BCL2* variant. Serial transplantation assays revealed that short-term treatment with the splicing modulator 17S-FD-895 reduced AML LSC burden and self-reneval in dose-dependent manner. Hence, this promising strategy of eradication of LSC by targeting key spliceosome component could be exploited therapeutically.

Another pladienolide B-derived splicing modulator H3B-8800 was shown to have selective cytotoxic effects on spliceosome-mutant cells, conceivably due to retention of GC-rich introns in pre-mRNAs encoding distinct spliceosome components [50]. Moreover, Sankar et al. [51] reported that inhibition of SF3b complex activity with H3B-8800 in ALL cells with *KMT2A* rearrangement results in differential splicing, which promotes the expression of γ-*H2AX* and reduces levels of *RAD51* and *FANCD2*, well-known markers of early response to the induction of DNA double-strand breaks. Notably, a similar effect was observed upon knockdown of a SF3b complex subunit *PHF5A* and *SF3B1 *[51]. In accordance with previous reports, a recent study indicates that the SF3b splicing complex plays a critical role in the regulation of DNA break repair, as depletion of its compounds hampered DNA end resection and homologous recombination [52]. Interestingly, H3B-8800 have entered phase I clinical trial in patients with AML, MDS and CMML (NCT02841540, study registration date: 2016-07-20) (Table 1) [53].

**Table 1:** Small-molecule modulators targeting spliceosome-related proteins in clinical trials

Trial identifier	Phase	Status	Target	Drug	Combination	Disease	Report from the study
NTC02841540	I	Active, not recruiting	SF3B1	H3B-8800 (RVT-2001)	NA	MDS, AML, CMML	To date, results have demonstrated dose-dependent target engagement (such as changes in mature mRNA transcripts), a predictable pharmacokinetics profile, and favorable safety profile, even with prolonged dosing. While no objective complete responses or partial responses were achieved, 14% of enrolled patients had decreased requirements for red blood cell or platelet transfusion [53].
NCT01692197	II	Completed	RBM39	E7070	Idarubicin, cytarabine	r/r AML, high-risk MDS	The recently presented results demonstrated favorable effects of combining indisulam with chemotherapy, as the estimated 1-year overall survival was 51% for responders in comparison to 8% for non-responders [54].
NCT02783300	I	Completed	PRMT5	GSK3326595	NA	Solid tumors and NHL	Preliminary data demonstrated partial responses in patient with HPV + cervical cancer and in 3 out of 14 patients with adenoid cystic carcinoma [55]. In part 2 dose expansion phase, 3/29 responses in patients with NHL were confirmed [56].

*NCT *ClinicalTrials.gov identifiers, *NA *Not applicable, *MDS *Myelodysplastic syndromes, *AML *Acute myeloid leukemia, *CMML *Chronic myelomonocytic leukemia, *r/r *Relapsed refractory, *SF3B1 *Splicing factor 3b subunit 1, *RBM39 *RNA binding motif 39, *PRMT5 *Protein arginine methyltransferase 5, *NHL *Non-Hodgkin’s Lymphoma

However, several mutations in SF3b subcomplex components that confer resistance to pladienolide, herboxidiene and H3B-8800 have been identified to date, including *PHF5A*^Y36C^, *SF3B1*^K1071E^, *SF3B1*^R1074H^, and *SF3B1*^V1078I48^.

Although SF3B1 inhibitors were demonstrated to block early steps of spliceosome assembly, Effenberger et al. [57] reported their role in later stages of the spliceosome catalytic cycle, such as exon ligation. Thus, consistent with previous studies, SF3B1 activity was shown to be required throughout the whole splicing process.

### Indirect splicing modulation

#### Splicing protein kinases

Effects of splicing inhibitors indirectly targeting distinct spliceosome components, including the SR-rich protein-specific kinases (SRKPs) and the dual-specificity Cdc2-like kinases (CLKs) are under investigation. For instance, a small-molecule pan-CLK inhibitor SM09419 was found to significantly downregulate Wnt signaling and inhibit proliferation in AML cells regardless of *FLT3* status [58]. Furthermore, treatment with CLK2 inhibitor T-025 significantly reduces CLK-dependent phosphorylation, thus promoting exon-skipping events, inducing apoptosis, and suppressing growth in both AML MV-4-11 and patient-derived xenograft models [59]. Notably, MV-4-11 cell line well as xenografts derived from AML patient presented the highest sensitivity to splicing modulator T-025 across all evaluated hematological cancer cells in this study. Intriguingly, Iwai et al. [59] characterized a synergistic apoptosis induced by MYC activation and T-025-mediated CLK inhibition, which might result from the concomitant perturbation of distinct splicing pathways.

#### Protein arginine N-methyltransferases

Another example of compounds that indirectly affect the spliceosome function are protein arginine N-methyltransferase-5 (PRMT5) and 1 (PRMT1). In physiological conditions, PRMT5 and PRMT1 are responsible for the methylation of a wide range of cellular proteins, including transcription factors, histones, and spliceosome components [60]. Previous studies revealed an oncogenic activity of the overexpressed PRMTs in distinct hematological malignancies, including AML (CARM1 [61, 62], PRMT5 [63]), diffuse-large B cell lymphoma (DLBCL) (PRMT5 [64]), and B-cell leukemia/lymphoma (PRMT5 [65, 66]).

While normal PRMT5 activity is crucial for the assembly of Sm proteins to the spliceosome [67], PRMT1 methylates RBM15, an RNA-binding protein which regulates splicing of multiple mRNAs, including those encoding proteins involved in hematopoiesis: *GATA1*, *RUNX1*, *TAL1* and *c-MPL* [68]. Notably, the downregulation of RBM15 induced by an increased expression of PRMT1 inhibits megakaryocyte terminal differentiation in acute megakaryocytic leukemia [68].

Recent studies indicate that targeting those splicing-affecting PRMTs might be a promising therapeutic approach for different hematological cancers. For instance, treatment of mantle cell lymphoma (MCL) cell lines with an inhibitor of arginine PRMT5 EPZ015666 (GSK3235025) resulted in cell death due to inhibited methylation of splicing-related protein SmD3 [69]. Furthermore, EPZ015666 reduced the growth of patient-derived MCL xenografts in a dose-dependent manner [69]. Of note, near 95% tumor growth inhibition after 21 days of oral dosing of EPZ015666 was reported in multiple MCL xenograft models, thus highlighting the potent anti-tumor activity of this PRMT5 inhibitor. To date, distinct mechanisms for PRMT5 in driving oncogenesis have been reported, including RNA processing and cell death, however, details on its transcriptional regulatory function requires further investigation. Fong et al. [70] observed that distinct inhibitors of PRMTs, such as PRMT5 selective inhibitor GSK3203591 and a pan-type I PRMTs inhibitor MS023, induce preferential killing of SF-mutated AML cells over wild-type control. According to the study by He et al. [71], PRMT1 contributes to FLT3-ITD + AML cells survival and growth in *FLT3*-methylation dependent manner. The effect of *FLT3* methylation on AML maintenance was found to be not solely dependent on PRMT1 activity, since the *FLT3* methylation persisted in leukemic cells after administration of kinase inhibitor. However, studies in patient-derived xenograft and murine AML models showed that the combination of tyrosine kinase inhibitor AC220 with MS023 increased apoptosis of FLT3-ITD + AML cells as compared to AC220 treatment alone [71].

Several clinical trials have investigated the effects of PRMT5 and PRMT1 inhibitors in hematologic malignancies, two of them, however, have been terminated due to either a relatively high incidence of thromboembolic events and lack of observed clinical efficacy (NCT03666988) or internal review of clinical data (NCT03614728).

#### Sulfonamides

To date, several studies identified molecular mechanisms underlying indisulam’s and related sulfonamides (tasisulam, chloroquinoxaline sulfonamide, and E7820) anticancer activity [72–74]. The presented results showed that indisulam forms a complex with the U2AF-related SF RBM39 (RNA binding motif protein 39), CAPER?? (co-activator of activating protein-1 and estrogen receptors), and the E3 ubiquitin ligase receptor DCAF15 (DDB1 and CUL4 associated factor 15), resulting in RBM39 polyubiquitination and proteasomal degradation. As RBM39 is involved in AS regulation, degradation of this nuclear protein leads to aberrant intron retention and exon skipping, and therefore to cytotoxicity in numerous cancer cell lines [72]. Of note, RBM39 was demonstrated to control AS of AML-related genes, such as *GATA2*, *HOXA9*, and *BMI1 *[75]. This study also revealed that hematopoietic and lymphoid malignancies with SF mutations are especially vulnerable to indisulam-induced cytotoxicity [75].

### SFs mutations and its impact on the activity of splicing-based drugs

Interestingly, it was demonstrated that leukemic cells harboring specific SF mutations display an increased sensitivity towards splicing modulators. For instance, *SRSF2*^P95H^ and *SF3B1*^K700E^ mutations causes sensitivity to the pladienolide B-derived spliceosome modulator E7107 [76, 77]. Moreover, E7107 has been found to render MDS cells with U2AF1^S34F^ mutation more sensitive to ATR inhibitors, since it induces augmented R-loop formation and elicits an R-loop-dependent ATR response [8]. Furthermore, Wang et al. [76] observed more pronounced splicing changes in AML cells with either *SF3B1*^K700E^, *SF3B1*^K700E^, *SF3B1*^K666N^, *SF3B1*^H662Q^, *SRSF2*^P95H^, or *U2AF1*^S34F^ mutations treated with arylsufonamide E7820. Seiler et al. [50] found that the H3B-8800-induced selective cytotoxicity to AML cell lines was greater in the presence of *SF3B1*^K700E^ mutation. Furthermore, both in vitro and in vivo studies revealed a particular sensitivity of leukemic cells with *U2AF1*^S34F^ mutation to sudemycin application in comparison to cells with *U2AF1*^WT^[78].

### AS modulators in combination therapy

Emerging evidence indicate the improved efficacy of splicing modulators in combination with selected therapeutic agents in blood neoplasms. A recent study demonstrated that a combination of pladienolide B and dexamethasone displays a synergistic antitumor effect in glucocorticoid-resistant pediatric ALL [42]. Since poor response to the initial GCs administration has been found to be linked with unfavorable outcomes in patients with ALL, the use of splicing inhibitors constitutes an attractive therapeutic option, which might improve the sensitivity of GC-resistant leukemic cells to combination chemotherapy including GCs.

Despite the remarkable clinical efficacy of Bruton kinase inhibitors (BTKi), such as ibrutinib, acalabrutinib, and zanubrutinib, distinct mechanisms of resistance have been characterized that lead to limited or no complete remissions in patients with CLL [79]. Consistent with this, cases with no BTK or downstream target mutations suggest that other signaling pathways might contribute to the maintenance and survival of leukemic cells in the lymph nodes. Since it was revealed that BTKi administration induces efflux of leukemic cells from nodal compartments into the bloodstream, where they can be killed by novel targeted drugs, various combination therapy approaches have been proposed [80, 81]. For instance, an enhanced antitumor effect of sudemycin in combination with ibrutinib was presented both in vitro and in xenograft models of CLL [82]. Xargay-Torrent et al. [82] reported that sudemycin exhibits its antitumor effect through splicing modulation of various genes involved in prosurvival pathways irrespective of *SF3B1* mutation status. Notably, sudemycin D6 decreased the presence of leukemic cells in the peripheral blood and and spleen of NOD/SCID/*IL2Rγ*-/- mice engrafted with primary cells from patients with CLL. The reduction of CLL cells in peripheral blood samples was near 50 and 40% for *SF3B1*^WT^ and *SF3B1*^MUT^ cases, respectively, while in the spleen – about 25 and 40% in *SF3B1*^WT^ and *SF3B1*^MUT^ cases, respectively. However, an enhanced cytotoxic effect of sudemycin in combination with ibrutinib was probably associated with exon 25 skipping of gene encoding the inhibitor of BTK (*IBTK*). IBTK is a key negative regulator of BTK, and its increased activity is related to a decreased expression level of pBTK. High expression of pBTK was found to be associated with enhanced sensitivity of AML cells to ibrutinib, therefore the synergic downregulation of BTK activity with sudemycin and ibrutinib combination in leukemic cells, identified by increased pBTK levels, might result from sudemycin-induced changes in IBTK splicing [82].

Moreover, a different combination therapy approach has been proposed. It was demonstrated that splicing modulator E7107 affects the apoptotic pathway in human and murine CLL cells, thus favoring the BCL2 dependence and providing the opportunity for combinatorial treatment strategies with BCL2 inhibitors [83]. Notably, a combination of venetoclax with E7107 enhanced the sensitivity of leukemic cells to the BCL2 inhibitor and overcame venetoclax treatment resistance in murine CLL models [83]. Furthermore, Larrayoz et al. [84] documented that splicing modulation combined with BCL2/BCL-XL antagonists ABT-263 or ABT-199 produces a significant reduction in CLL cell viability in pro-survival conditions provided by IL4/CD40L stimulation. Interestingly, in both studies, splicing modulation which reprogrammed the apoptotic dependence in CLL, was induced independently of *SF3B1* mutation status. Indeed, proteomic studies revealed a significant enrichment in spliceosome components in CLL samples as compared to healthy volunteers, indicating the irrelevance of *SF3B1* mutation status in splicing disruption [85].

Comprehensive RNA-seq studies have identified profound changes in splicing profiles between samples collected from multiple myeloma (MM) patients and healthy individuals [86]. Of note, these splicing aberrations were found to be implicated in the overall clinical outcome of patients with MM. Interestingly, *SF3B1* loss-of-function mutations are observed in ~ 2% of MM cases, which suggests that splicing alterations are triggered by some other events [87, 88]. Studies on MM xenograft murine models showed that low dose of splicing modulators, including sudemycin and meayamycin, also shifts apoptotic dependence in MM cells, thus enhancing their sensitivity to venetoclax, irrespectively of SFs mutations, MYC expression level, and t(11;14) [89]. Although the frequency of SF mutation in MM is relatively low, a potent anti-tumor activity of splicing modulators was found to be linked with a remarkable change in the *MCL1* spliced variants usage in treated cells than controls. As a result, MM cells display increased activity of BCL2, which supports the synergistic activity observed in combination with the BCL2 inhibitor venetoclax.

Despite accumulating reports, the details of the mechanisms of antitumor effects of splicing modulators remain largely unknown. It has been speculated that the preferential tumor cytotoxicity of splicing modulators might be the consequence of differential splicing of a subset of expressed genes involved in cell cycle regulation and apoptosis, which presumably contain weak 3’ splice sites [46, 90]. Besides their concentration and affinity for SFs, pre-mRNA splicing efficiency depends also on competing pre-mRNAs. A study on yeast performed by Munding et al. [91] revealed that splicing is less efficient when ribosomal protein genes are expressed and markedly improved globally upon repression of these genes. Therefore, weakly competitive pre-mRNAs produced by genes involved in cell cycle regulation and programmed cell death might be more vulnerable to splicing inhibitors. In accordance with this report, MYC hyperactivation noticeably increases total mRNA synthesis, and, in turn, the burden on the spliceosome [92]. Notably, MYC-dependent cancer cell lines and animal models were found to be significantly more sensitive to genetic or pharmacological SF3B1 inhibition than tumor cells in the normal MYC state [92]. As various splicing modulators, particularly globally acting ones, might also affect non-malignant cells, efforts should be made to identify a therapeutic approach, which would alleviate the toxicity that was observed in clinical trials.

### Antisense oligonucleotides

Advances in molecular biology provide a new opportunity to revisit therapeutic strategies for splicing modulation. To date, numerous preclinical studies have highlighted the capabilities of antisense oligonucleotides (ASOs) to induce antitumor effects (Table 2).

**Table 2:** The preclinical investigation of the antisense strategies based on modified oligonucleotides

Target	Experimental model	ASO	Biological outcome following ASO-based treatment	References
*CD5*	PBMCs from CLL patients, Jurkat cell line	anti-*CD5* PNA	• CD5 down-modulation • Enhanced drug-induced apoptotic response mediated by co-treatment with fludarabine	[93]
*BCR-ABL*	CML-derived cell lines BV173 and K562	Naked *BCR-ABL*-targeting antisense double-stranded DNA olignonucleotide	• Reduction of BCR-ABL levels in dose-dependent manner and suppress of proliferation of BCR-ABL-positive CML cells	[94]
*IRF4*	Human myeloma cell line H929 and patient-derived xenograft model	*IRF4*-targeting ASO	• Impediment of tumor formation and myeloma regeneration through disruption of cell cycle progression and sensitization to myeloma drugs • Reduction of c-MYC expression • ION251 has entered a phase I clinical trial in patients with r/r MM (NCT04398485)	[95]
*STAT3*	AML cell lines KG1a, KT-1, CMK, MOLM13, MOLM14, NB4, MV411, MDS-L, and U937, patient-derived hematopoietic cell populations, patient-derived xenograft model	*STAT3* ASO (AZD9150)	• Disruption of leukemic cell growth in vitro and in vivo • Increase of hematopoietic differentiation • Down-modulation of the *MCL1* gene as well as other oncogenic genes expression	[96]
*miRNA-19a*	HL60 cell line	*miRNA-19a* ASO	• Inhibition of leukemic cell proliferation and increase of apoptosis • Synergistic anti-tumor effect with Ara-C	[97]
*BIM*	K562 and KCL22 cell lines	Splice-switching *BIM*-targeting ASOs (ASO-13, ASO-15, ASO-18, ASO-28, ASO-29, ASO-33, ASO-52, ASO-53)	• Enhancement of pro-apoptotic BIM protein variant expression and restoration of the imatinib-induced apoptosis and TKI sensitivity via increase of the inclusion of BIM exon 4 in CML cells with the TKI resistance-related *BIM* deletion polymorphism in intron 2	[27]
*WT1*	HL-60 and K562 cell lines	ASO*WT1*^exon5^	• Increase of the exon 5-skipped *WT1* isoform production, which enhance the proapoptotic signal via upregulation of WT1 target thrombospondin 1	[98]
*TdT*	Molt-4 cell line	*TdT*-targeting antisense PNA	• Increased rate of apoptosis mediated by TdT downregulation through inclusion of intron 7 followed by exon 7 skipping in the TdT gene	[99]

*PBMC *Peripheral blood mononuclear cell, *CLL *Chronic lymphocytic leukemia, *PNA *Peptide nucleid acid, *CML *Chronic myeloid leukemia, *IRF4 *Interferon regulatory factor 4, *ASO *Antisense oligonucleotide, *MM *Multiple myeloma, *NCT *ClinicalTrials.gov identifier, *STAT3 *Signal transducer and activator of transcription 3, *AML *Acute myeloid leukemia, *MCL1 *Myeloid leukemia 1 gene, *Ara-C *Cytarabine, *BIM *Bcl-2-like protein 11, *TKI *Tyrosine kinase inhibitor, *TdT *Terminal deoxynucleotidyl transferase

**Fig. 3:**
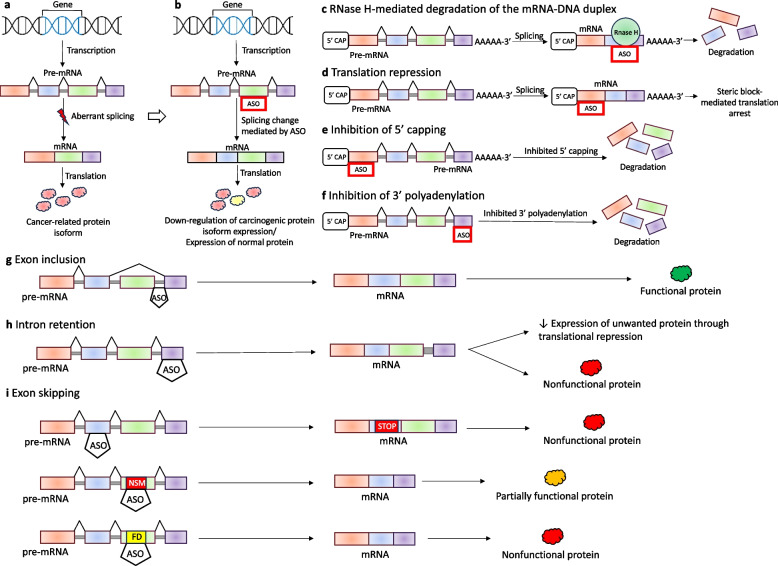
The role of ASOs in aberrant splicing correction. **a** Disruption of the splicing process might result in the formation of aberrant splice isoforms, which are translated into carcinogenic protein variants. **b** ASO-induced splice-switching can downregulate the production of carcinogenic protein variants by correcting missplicing. ASOs can modulate gene expression also through (**c**) RNAse H-mediated cleavage of the specific mRNA-DNA duplex, translation repression (**d**), and inhibition of either (**e**) 5’ capping or (**f**) 3’ polyadenylation. ASOs-mediated modulation of splicing include exon inclusion (**g**), intron retention (**h**), and exon skipping (**i**). Therefore, ASOs can be designed to enhance the generation of a specific protein or restore a specific exon in the disease-causing exon-skipped form of the gene. Exon-skipping strategy can be used to induce premature stop codon, remove an exon containing a nonsense or frameshift mutation, or remove an exon encoding crucial functional domains. ASO – antisense oligonucleotide, STOP – premature stop codon, NSM – nonsense mutation, FD- functional domain

ASOs are a complementary nucleic acid fragments that bind to specific splice sites or splicing regulatory elements, thus correcting aberrant splicing events (Fig. 3) [100].

ASOs mechanisms of action include splice site switching, alteration of splicing to include/exclude selected introns/exon, and induction of exon-skipping events.

It was also observed that ASOs might modulate RNA function by correcting cryptic splice sites [100]. Furthermore, ASOs influence the protein translation in cells via two distinct mechanisms [101]. One of them is based on the interaction between ASOs and either mRNAs or miRNAs, which, in turn, selectively changes the translation of proteins. ASOs might also bind directly to the 5’-UTR regulatory ORF or stem-loop structure sequences of selected proteins, thus increasing the expression of specific targets. In the second case, ASOs might induce mRNA cleavage by endogenous nucleases, including RNase H1 and argonaute 2.

Over the past years, the clinical efficacy of ASO-based therapeutic strategies has been evaluated in solid tumors. Nevertheless, distinct ASOs are being investigated for use to enhance the effectiveness of standard anticancer therapy for the treatment of hematological disorders.

#### *BCL2*-targeting ASO

G3139 is an 18-mer phosphorothiolate (PS)-modified ASO that targets the first six codons of the *BCL2* mRNA and induces the RNAse H1-dependent mRNA degradation. In monotherapy, G3139 (oblimersen) failed to improve outcomes in patients with CLL (NCT00021749) [102]. However, oblimersen has demonstrated promising synergistic effects in combination with standard chemotherapy in patients with CLL and B-cell NHL [103–105]. The effectiveness of fludarabine and cyclophosphamide with or without oblimersen was assessed in a phase III clinical trial in patients with relapsed or refractory CLL [103]. The results demonstrated that the proposed combination therapy significantly increased the complete response rate in patients with r/r CLL. A 5-year follow-up of this trial demonstrated that the addition of G3139 to fludarabine and cyclophosphamide remarkably extended survival in patients who achieved a partial response or better (NCT00024440) [104]. In another phase II clinical trial, the combinational effect of G3139 and rituximab was evaluated in patients with recurrent B-cell NHL, showing a good safety profile and beneficial effect in the subgroup of patients with indolent disease (NCT00054639) [105]. The efficacy of G3139 in combination therapy in patients with DLBCL is being evaluated in phase I/II clinical trials (NCT00070083, NCT00086944).

The results of a phase II trial in patients with relapsed multiple myeloma (MM) demonstrated that the combination of G3139, dexamethasone, and thalidomide is well tolerated, and response rates are promising (NCT00049374) [106]. However, the results of a phase III trial in patients with relapsed MM demonstrated no significant differences between groups receiving dexamethasone with or without G3139 relative to time to tumor progression (NCT00017602) [107].

Previous reports from clinical trials indicated that G3139 might be effective in combination with chemotherapy in patients with AML [108, 109]. However, recent evidence from a phase III clinical trial demonstrated that the addition of G3139 to chemotherapy failed to improve outcomes of previously untreated older AML patients (no differences in overall survival, disease-free survival (DFS), or event-free survival) (NCT00085124) [110]. Nevertheless, an improved DFS was observed in patients with secondary AML.

Also in another phase II trial, the combination of G3139 and imatinib was administered to patients with imatinib-resistant CP CML (NCT00049192). Despite good safety profile, the study showed no clinical benefit from G3139 addition to imatinib in the treatment of CML [111]. The failure of *BCL-2*-targeting ASO-based therapy might be associated with a long protein half-life. Nevertheless, the inhibition of BCL2 antiapoptotic BH3 domains with small molecule inhibitors, such as venetoclax, has revealed significantly improved outcomes in patients with B-cell malignancies [112].

#### *GRB2*-targeting ASO

BP1001 is a dioeoylphosphatidylcholine lipid-encapsulated P-ethoxy-modified ASO that was designed to block the expression of the growth factor receptor-bound protein 2 (*GRB2*) and, in turn, suppress the activation ERK1 and ERK2 and leukemia progression. A phase I study showed good safety profile and anti-leukemic activity in patients with refractory or relapsed AML and high-risk MDS (NCT01159028) [113]. Furthermore, a significant reduction of peripheral blood and BM blast count in AML patients administered BP1001 alone or in combination with low-dose cytarabine. The effectiveness of BP1001 in combination with venetoclax and decitabine is now being evaluated in a phase IIa trial in AML patients (NCT02781883). Moreover, a phase I study currently investigates the safety and tolerability of another *BCL-2*-targeting ASO BP1002 in patients with advanced lymphoid malignancies (NCT04072458).

#### *STAT3*-targeting ASO

Previous study revealed upregulation of STAT3 in the activated B-cell-like DLBCL cells [114]. Scuto et al. [115] demonstrated that inactivating STAT3 reduces growth of DLBCL patient-derived xenografts in a preclinical model. Results of this study demonstrated that even partial downregulation of STAT3 in activated B cell-like DLBCL could suppress tumor growth. It was shown that tumor regression in STAT3 shRNA lentivirus Ly3-bearing mice was associated with Caspase-3-dependent apoptosis and significant decrease of STAT3 target genes encoding MCL1, C-MYC, and survivin, as well as inhibition of tumor-promoting environment. AZD9150/danvatirsen is a 16-mer ASO triggers the RNAse H1-mediated cleavage of the *STAT3* mRNA, thus downregulates its expression in cells. Danvatirsen contains ten PS-modified deoxynucleotides flanked on each end by three constrained ethyl (cEt) nucleotides. Results of a phase Ib trial demonstrated the safety and tolerability of danvatirsen in DLBCL as well as a promising efficacy of *STAT3* ASO with two complete responses and two partial responses in patients with DLBCL (*n* = 30) (NCT01563302) [116]. Other phase I/Ib clinical trials are evaluating the safety and tolerability of acalabrutinib in combination with either STAT3 inhibitor danvatirsen, ATR inhibitor AZD6738, anti-CD47 antibody Hu5F9-G4 plus anti-CD20 antibody rituximab, or bromodomain-containing protein 4 (BRD4) inhibitor AZD5153 in patients with r/r DLBCL (NCT03527147). Early report from this trial showed that the overall response rate was 24% for DLBCL patients (*n* = 17) who were administered combination treatment with BTK inhibitor and ani-STAT3 oligonucleotide [117]. Nevertheless, limited clinical benefit of simultaneous targeting of STAT3 and BTK was observed in this small r/r DLBCL group.

Phase Ib study has evaluated the safety and efficacy of the anti-programmed death ligand 1 (PD1) antibody durvalumab in combination with danvatirsen or an ani-cytotoxic T-lymophocyte-associated antigen 4 (CTLA-4) antibody tremelimumab in DLBCL (NCT02549651). Although the results of this trial showed that durvalumab can be administered safely in combination with danvatirsen or tremelimumab, the anticancer activity was limited [118].

Furthermore, danvatirsen in combination with venetoclax has entered a phase I trial in patients with r/r MDS and AML (NCT05986240).

#### *C-MYB*-targeting ASO

The PS-modified 24-mer ASO G4460/ODN targets codons 2 to 9 of the *c-MYB* mRNA and downregulates the activity of C-MYB transcription factor. Phase II study evaluated the efficacy of *c-MYB*-targeting ASO in combination with chemotherapy and subsequent bone marrow transplantation in patients with CML (NCT00002592). Results of this study demonstrated limited therapeutic activity of *C-MYB* ASO [119].

#### *TP53*-targeting ASO

The clinical investigation of a *TP53*-targeting ASO cenersen (either alone or in combination with chemotherapy) has been evaluated in a phase I trial in patients with MDS (NCT02243124), as well as phase II for lymphoma and AML (NCT00967512, NCT00074737, NCT00636155). Cenersen is a 20-mer phosphorothiolate ASO that binds exon 10 of the *TP53* mRNA, which results in catabolism of *TP53* mediated by RNAse H1. However, the reported data indicated that cenersen administration has rather weak therapeutic effect [120, 121].

#### *XIAP*-targeting ASO

Since overexpression of the X-linked inhibitor of apoptosis protein (XIAP) in AML might confer chemoresistance, molecules that downregulates XIAP were thought to be useful for the treatment of patients with high levels of this antiapoptotic protein. AEG35156 is a 19-mer ASO with eleven deoxynucleotides flanked by 4 2’-O-methyl-modified RNA bases on both 5’ and 3’ ends. Despite promising preclinical results relative to cell death induction and sensitization of cell to chemotherapy, AEG35156 in combination with chemotherapy failed to improve rates of remission in patients with AML [122, 123].

#### *RNR*-targeting ASO

In a phase I trial adults (=60 years) with r/r AML GTI-2040, a 20-mer ASO complementary to the R2 subunit mRNA of ribonucleotide reductase (*RNR*), in combination with high dose cytarabine. Since nucleoside analog chemoresistance might result from RNR overexpression, downregulation of RNR might potentially enhance cytotoxicity by favoring cytarabine-CTP DNA incorporation. Despite the good safety profile of GTI-2040/HiDAC, no complete remissions were observed in this study [124].

## Conclusions

In this Review, we have highlighted the functional effect of aberrant splicing on therapy resistance and summarized current knowledge about novel splicing-targeting drugs. The insensitivity to targeted therapies might result from specific splicing events, such as exon skipping and intron retention, which lead to the production of aberrant isoforms lacking drug-targeting domains. Moreover, mis-splicing was reported to be implicated in the process of antigen escape in B-cell malignancies treated with antigen-targeted immunotherapies, including CAR T-cell approach.

Although immunotherapy remains more effective for hematologic malignancies than for solid tumors, it is worth mentioning that mis-splicing events might help in the development of antigen-based immunotherapy for solid neoplasms. Recent studies have identified numerous tumor-specific peptides produced in distinct solid tumor types [125]. Interestingly, SFs dysfunction-induced global splicing alteration might generate more neoantigens than somatic single nucleotide variants in various tumor types, including breast and ovarian cancers [2]. Since dysregulation of SFs contributes to an enhanced immunogenicity, it was suggested that formulation of immunotherapy and SF modulation combinations might improve treatment outcomes in patients with solid tumors. To date, several clinical trials (NCT03854227, NCT04196257, NCT03394144, NCT00357747, NCT00372736) have evaluated the tolerability and efficacy of splicing modulators (e.g. PF-06939999, BP1001, AZD9150, and AEG35156) in monotherapy or in combination with chemo- or immunotherapy. Emerging reports unveils the implications of AS disruption on immunogenicity modulation and describes challenges to immunotherapy that are specific for solid malignancies [126–128].

Given the low conservation of AS events observed in humans and mice (20–25%) [129], it seems crucial to develop human organoids or humanized animal models to investigate the role of aberrant splicing in cancer development and therapy resistance. Emerging data showed that ASO-induced splice-switching strategy can be effectively used in combination with chemotherapy to modulate the expression of targeted genes, thus improving the outcome of patients with hematological disorders. However, limited data is available on phase III trials evaluating the clinical efficiency of ASOs in blood neoplasms. Noteworthly, there are several medical examples of the use of ASOs, including nusinersen – an ASO that induces splicing correction, which was developed for the treatment of spinal muscular atrophy. The ASO strategy has also been successfully applied for the restoration of the reading frame as a therapy for patients with Duchenne muscular dystrophy.

Taking into consideration that stability and site-specific delivery efficiency are the major challenges of targeting aberrant splicing events/unwanted mRNA variants, efforts into chemical modifications should be made to improve the efficacy and safety of ASOs. In a view of a relatively high prevalence of SF mutations in hematological disorders, further research is needed to provide a comprehensive view on AS regulation in leukemic cells, and to evaluate the effectiveness of ASO-based therapy approaches to correct aberrant splicing.

## Data Availability

No datasets were generated or analysed during the current study.

## Abbreviations

AML: Acute myeloid leukemia
AS: Alternative splicing
ASO: Antisense oligonuleotides
B-ALL: B-cell acute lymphoblastic leukemia
BTKi: Bruton kinase inhibitors
CAR-T cells: Chimeric antigen receptor-modified T cells
CLK: Cdc2-like kinases
CLL: Chronic lymphocytic leukemia
CML: Chronic myeloid leukemia
CMML: Chronic myelomonocytic leukemia
dCK: Deoxycytidine kinase
DLBCL: Diffuse-large B cell lymphoma
ESE: Exonic splicing enhancer
ESS: Exonic splicing silencer
FPGS: Folypoly-γ-glutamate synthetase
GC: Glucocorticoid
ISE: Intronic splicing enhancer
ISS: Intronic splicing silencer
LSC: Leukemia stem cell
MCL: Mantle cell lymphoma
MDS: Myelodysplastic syndromes
MM: Multiple myeloma
MTX: Methotrexate
NMD: Nonsense-mediated decay
SF: Splicing factor
snRNP: Small nuclear ribonucleoprotein
SR: Serine and arginine-rich proteins
TKI: Tyrosine kinase inhibitor
